# Preparedness of the Healthcare Personnel Against the Coronavirus Disease 2019 (COVID-19) Outbreak: An Audit Cycle

**DOI:** 10.3389/fpubh.2020.00502

**Published:** 2020-09-11

**Authors:** Nowera Zafar, Zohaib Jamal, Muhammad Mujeeb Khan

**Affiliations:** Department of Infectious Diseases, Rawalpindi Medical University and Allied Hospitals, Rawalpindi, Pakistan

**Keywords:** coronavirus, COVID-19, healthcare personnel, international guidelines, Pakistan, preparedness

## Abstract

With the increasing spread and mortality of the COVID-19 (Coronavirus disease 2019) pandemic, it is essential for the healthcare community to be prepared per the international standards. This study is focused on assessing the preparedness of healthcare personnel and the effectiveness of an educational intervention to improve this preparedness in those dealing with the COVID-19 infection. A prospective, multicenter audit cycle was conducted on 400 healthcare professionals (271 junior doctors, 90 nurses, 39 non-clinical hospital workers) sampled through stratified random sampling. A questionnaire that was based on “Centers for Disease Control and Prevention (CDC's) checklist for healthcare personnel's preparedness for transport and arrival of patients with confirmed or possible COVID-19” was sent to the participants after which an informative document, framed on the information provided by World Health Organization (WHO), CDC, and local guidelines from the Government of Pakistan's website, was distributed through social media platforms. The questionnaire was repeated after 2 weeks to close the audit loop. Chi-Square test and paired sample *t*-test were used to test significance. In the pre-intervention portion of the study, it was found out that the doctors and nurses had higher knowledge scores compared to the non-clinical hospital staff (*p* ≤ 0.05). A statistically significant improvement was seen after the educational intervention was deployed (*p* ≤ 0.05). The study concludes that the non-clinical staff, being a vital part of the healthcare framework, need to be educated and effective approaches for official inclusion of relevant information need to be incorporated into clinical practice to limit the transmission of COVID-19.

## Introduction

Coronavirus disease or COVID-19 is a newly discovered infectious ailment of the respiratory tract that was first reported in the province of Wuhan, China in December of 2019 and since then, has spread rapidly across the globe (1). The causative agent belongs to the species of Severe acute respiratory syndrome-related coronavirus, which has been given the name of Severe Acute Respiratory Syndrome Coronavirus-2 (SARS-CoV-2) by the International Committee on Taxonomy of Virus (2). According to the World Health Organization, the total number of confirmed cases in the world has escalated up to 6,194,553 cases with 376,320 deaths, as of 2nd June, 2020 (3). The first case in Pakistan was confirmed on 26th February 2020 by the Ministry of Health and since then, an alarming number of people have been tested positive with a total of 76,398 confirmed cases and 1,621 deaths, as reported on 2nd June, 2020 (3–5). Given the high transmission of SARS-CoV-2 and increasing mortality, as indicated by the continuously rising curve in the graphs reported by the official website of the Government of Pakistan, the health care personnel of the country need to be adequately prepared to deal with the increasing numbers, similar to the rest of the world (6). Particularly significant is the preparedness of the junior doctors because a major portion of the decision making is handled by them, of the nurses as they are directly involved in patient care, and of the non-clinical hospital staff workers who are responsible for a great deal of movement within the hospital. Additionally, since they directly deal with the possible or confirmed cases, hence are most vulnerable to getting infected themselves and consequently, may become a source of transmission to thousands of the uninfected population. Therefore, the burden would be immense if a healthcare provider contracts the disease, making it highly necessary for them to be sufficiently prepared (7).

The Centers for Disease Control and Prevention (CDC) has laid out a checklist in reference to the preparedness of the healthcare personnel (HCP) to standardize care geared toward controlling the pandemic (8). Limited literature is available on how well-equipped the Pakistani medical community is to tackle the situation in line with these global standards and little is known about the validity of knowledge the healthcare professionals have on the matter.

Keeping in view the rapidly rising number of cases, this study is aimed at evaluating the preparedness of the healthcare professionals as per the international guidelines, finding out if a statistically significant difference is present between the preparedness of healthcare workers based on their designation, and if an improvement could be made through an educational intervention.

## Materials and Methods

The study is a prospective, multi-center, audit. Ethical approval was obtained from the Institutional Research Forum of Rawalpindi Medical University and allied hospitals. Data were collected from the three major government hospitals of Rawalpindi; Benazir Bhutto Hospital, Holy Family Hospital, and District Headquarters Hospital, Rawalpindi. In accordance with the Centers for Disease Control and Prevention's (CDC) definition of health care personnel (HCP), those serving in the frontline who had an increased risk of exposure, whether direct or indirect, were included in the study (9). This entailed the junior doctors (postgraduate trainees, medical officers, and house officers), nurses, and the non-clinical hospital staff (for e.g., ward clerks, technicians, maintenance workers, and security workers), among whom, those who had a working experience of 6 months or more were included. The HCP who had worked in a healthcare facility for <6 months and the laboratory personnel were excluded from the study. A pilot study based on 115 individuals was carried out to find out the estimated population proportion. Using the WHO sample size calculator, with the confidence level of 95%, anticipated population proportion 0.53 as per the pilot study, and absolute precision of 0.05, the sample size came out to be 383. A semi-structured, self-administered questionnaire, based on the “CDC's checklist for healthcare personnel preparedness for transport and arrival of patients with confirmed or possible COVID-19,” was sent to the participants via email and social media platforms such as WhatsApp. This was done to ensure safety protocol of social distancing, in light of the pandemic. An Urdu version was translated for the personnel not able to understand English, and after the pilot study, it was revised and modified by a subject specialist of each language. The questionnaire was also reviewed by a consultant of Infectious Diseases Department of Holy Family Hospital to ascertain its validity before proceeding. The final version contained 31 questions that assessed knowledge as well as the observance of safe practices and preventive measures. Participants were also asked if they considered themselves prepared for the management of the patients with COVID-19. The Public Health section of the Government of United Kingdom has provided a detailed guidance on the descriptive studies involving a clinical audit. In conformity with the methods suggested, the stages of clinical audit were followed which included preparation, criteria selection, performance measurement, devising a plan for improvement, and quantifying the results of the used method (10). A total of 400 people were included in the study through a stratified random sampling technique, the strata being formed on the basis of designation (doctors, nurses, and the non-clinical workers). A statement highlighting the intent of the study with the assurance of complete anonymity and confidentiality was added at the start. Individual responses were recorded only from those participants who had given informed consent via the option provided. Immediately after, as an educational intervention, an informative document, that was short and pertinent, was circulated through social media, which was purposed to be used as a quick reference guide and a learning tool. The material was extracted from CDC's “interim infection prevention and control recommendations for COVID-19 in health care settings,” the Government of Pakistan COVID-19 website for the local guidelines, and the World Health Organization. In order to close the audit loop, a re-audit was conducted after 2 weeks. The participants were contacted through the same method again, using emails and WhatsApp, and were sent reminders after 3 days of no response. Three hundred ninety three people participated in the second round, with a drop-out rate of 1.75%. The statistical computer package, IBM SPSS, Version 22 was used to analyze the data. Descriptive statistics were calculated and the Chi-Square test was used to find out the statistical significance of variables between the designation groups (doctors, nurses, and the non-clinical staff). A paired sample *t*-test was applied to test significance between the mean percentages of the correct answers and positive responses before and after the intervention (*p* ≤ 0.05 was considered statistically significant).

## Results

Table 1 represents the demographic characteristics of the 400 frontline health care personnel who participated in the study. The mean age of the respondents was 27.09 ± 3.7 in years. When it came to experience, more than 50% of the respondents, being the young frontline health care professionals, had ≥2 years of experience. Among the 271 front-line doctors, 159 were house officers, 35 were medical officers, and 77 were post-graduate trainees). Most healthcare professionals were working in the Wards (183; 45.75%) while the Isolation, where the suspected cases were kept, had the lowest number of staff working there (14; 3.5%).

**Table 1:** Demographic characteristics of the healthcare professionals.

**Demographics**	**Frequency (*n*)**	**Percentage (%)**
**Gender**		
Male	192	48
Female	208	52
**Designation**		
Doctors	271	67.75
Nurses	90	22.50
Non clinical hospital staff	39	9.75
**Working station**		
Ward	183	45.75
Emergency	122	30.5
OPD	32	8
ICU/CCU	24	6
Filter flu clinic	25	6.25
Isolation	14	3.5
**Hospital**		
Holy family hospital	185	46.25
Benazir Bhutto hospital	113	28.25
District headquarters hospital	102	25.25

Table 2 reveals the key highlights asserted by the Centers for Disease Control and Prevention (CDC) for preparedness of healthcare workers against COVID-19, and the corresponding percentages have been obtained based on the responses of the groups of doctors, nurses, and the non-clinical hospital workforce, for every questionnaire item. In the pre-intervention audit, it appeared that overall, the doctors and the nurses scored higher as compared to the non-clinical workers, where a sharp decrease in the percentages of well-informed respondents was noted. This was particularly noticeable for most of the knowledge questions about the disease itself and the protocols to follow, the differences being statistically significant (*p* ≤ 0.05). The questionnaire items on safe practices that showed a significant difference between the designation groups were those that inquired about the healthcare professionals' compliance with source control measures and their experience with a disease outbreak. However, surprisingly, in these two categories, the nurses and the supporting staff outnumbered the doctors. Overall, the knowledge questions were better answered than those about adherence to preventive measures. Noteworthy was that only about 30%, in all the groups and in total, responded positively when it came to observing source control measures, and awareness and participation in COVID-19 preparedness programs. In addition, fewer than half of the participants in each group considered themselves prepared for dealing with the pandemic.

**Table 2:** Centers for Disease Control and Prevention (CDC)'s preparedness checklist for health care professionals and the participants' response, pre-intervention, and post-intervention.

**CDC preparedness checklist**	**Percentages of correct answers**						**Difference between percentages of correct answers/improvement (%)**
	**Pre-intervention**					**Post**	
	**(%)**					**intervention (%)**	
	**Doctors %**	**Nurses %**	**Supporting**	***p*-value**	**Total**		
			**hospital**				
			**staff %**				
Knowledge about signs and symptoms	99.6	100.0	89.7		98.8	100	+1.2
Knowledge about diagnostic testing	85.6	68.9	25.6	**0.000**	76.0	98	**+22**
Knowledge about case definitions	72	22.2	5.1	**0.000**	54.3	76.3	**+22**
Knowledge about assessment and triage	64.9	17.8	5.1	**0.000**	48.5	74.5	**+26**
Knowledge about patient placement	78.2	55.6	0	**0.000**	65.5	84.3	+18.8
Knowledge about precautions	93	96.7	28.2	**0.000**	87.5	97.5	+10
Know how to donn PPE	52.4	56.7	20.5	**0.004**	50.3	92	**+41.7**
Know how to doff PPE	49.8	55.6	15.4	**0.002**	47.8	65	+17.2
Knowledge about PUI (person who needs to be investigated further)	94.5	100.	61.5	**0.000**	92.5	99.5	+7
Precautions for Aerosol Generating Procedures	85.6	91.1	20.5	**0.000**	80.5	67.5	−13
Knowledge about reporting	80.4	90.0	48.	**0.000**	79.5	94.3	+14.8
Knowledge of protocol in case of exposure	90.8	96.7	66.7	**0.000**	89.8	97.5	+7.7
Knowledge of protocol in case of illness	98.5	100.0	82.1		97.3	99.5	+2.2
Knowledge about how to contact state	86.7	91.1	64.1	**0.000**	85.5	100	+14.5
Usage of sanitizer/hand washing	96.3	100.0	100.0		97.5	100	+2.5
Following visitor management and exclusion	30.3	25.6	46.2	0.063	30.8	50.3	+19.5
Observance of source control measures	30.6	27.8	48.7	**0.050**	31.8	43.5	+11.7
Experience with a disease outbreak	46.1	73.3	59.0	**0.000**	53.5	63.3	+9.8
Awareness about COVID19 preparedness programs	23.2	30.0	17.9	0.271	24.3	58.8	**+34.5**
Participation in COVID19 preparedness programs	14.0	13.3	17.9	0.775	14.3	25	+10.7
Consider themselves prepared for COVID19	41.7	34.4	28.2	0.172	38.8	81.5	+42.7

*Bold values p ≤ 0.05 was considered statistically significant. Bold P-values were included only for those items where statistical analysis showed ≤ 20% cells with expected count <5*.

The usage of sanitizer was highly prevalent among doctors, nurses, and maintenance workers, with <3% being unable to use which they stated was because of unavailability. In contrast, about 70% of the HCP were working at the duty stations where visitor management and exclusion was not being regulate 62.6% attributed this to the absence of such guidelines while the other 37.4% believed that the manpower was not there to ensure the observation. Furthermore, among approximately three-quarters of the respondents not following measures to control the spread of infection from the source, ~30% mentioned the cause to be a shortage of masks, around 4% stated that there were not enough rooms to allow isolated examinations at a safe distance, while the rest (67%) believed both were the reasons.

Table 2 also shows that the performance of the health care professionals enhanced in almost all categories for their preparedness, in their post-intervention answers. The participants' practice of following preventive measures for AGPs, however, fell by 13%, where a separate room for performing the procedures was considered unnecessary by the respondents responsible for the decrease. Overall, the most poorly performed safety measures remained adherence to the rules to avoid unnecessary visitors (50.3%), following measures to control spread from the source (43.5%) and participation in the COVID-19 preparedness programs (25%).

Paired sample *t*-test showed a higher average percentage of participants who were following the CDC checklist for preparedness after the intervention. The statistical significance of this increase is shown in Table 3.

**Table 3:** Paired sample *t*-test for the comparison of means of pre-intervention and post-intervention correct answers/positive responses.

***P*-value**	**Mean**	**Standard**	***t***	**df**
		**deviation**		
0.000	−15.4047	13.4332	−5.255	20

**Figure 1:**
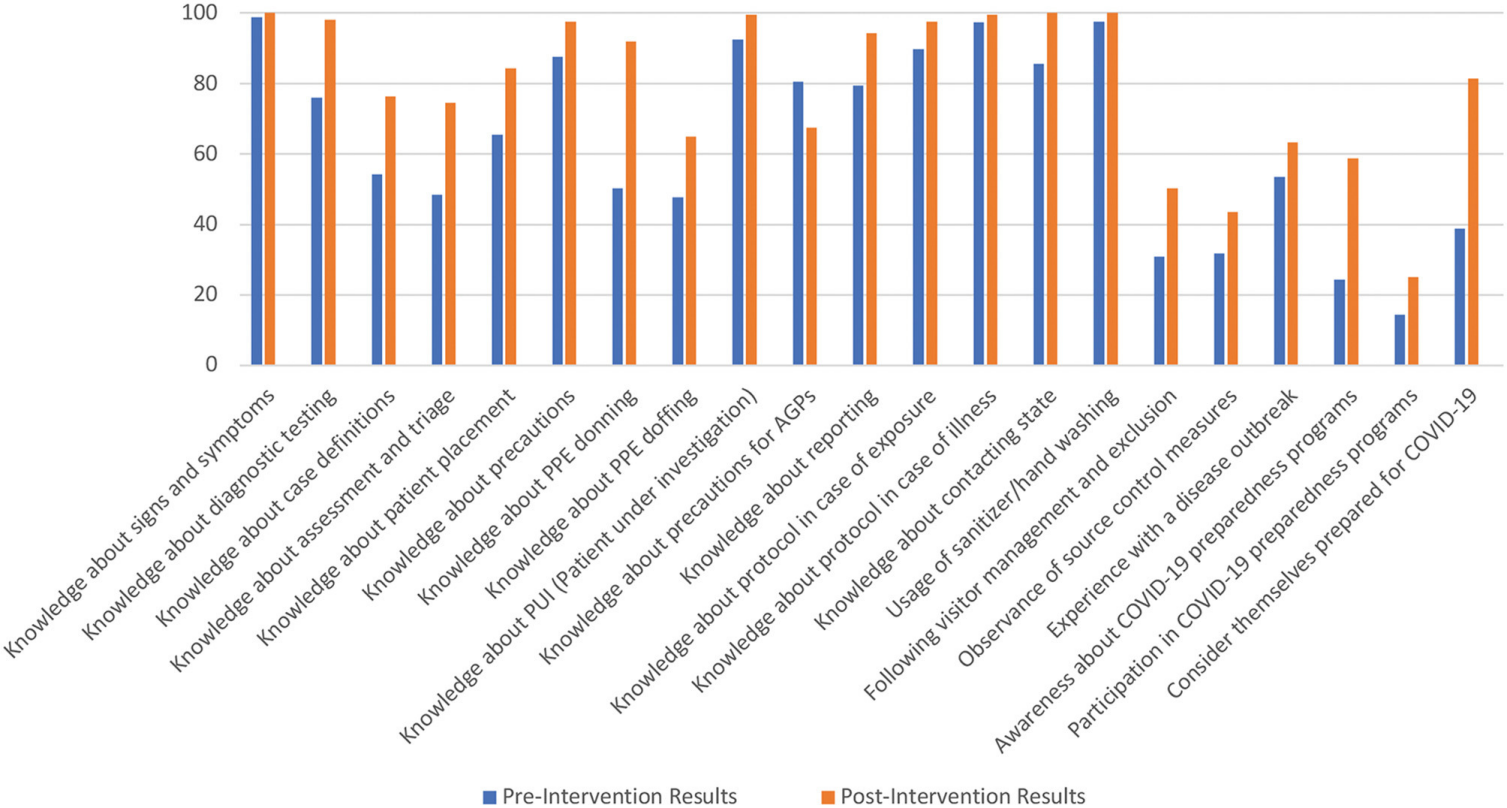
Increase in the percentages of correct answers and positive responses.

Figure 1 shows a graphical representation of the improvement observed.

## Discussion

The study was aimed at finding out the preparedness of the frontline healthcare professionals of Pakistan compared to the standards set by CDC. The audit cycle, which was performed in an attempt to find out if an educational intervention could bring an improvement in this preparedness, showed substantial improvement after the intervention (*p* = 0.000). In view of the importance to ensure social distancing, usage of social media messaging services was preferred over the face-to-face methods of holding workshops and lectures (11). Moreover, the pandemic has brought along with an added workload with tremendous physical and mental pressure for the healthcare community and consequently, as evident by the results in the study, participation in learning programs on preparedness was much lower compared to the awareness (12). Thus, such an information guide was feasible for the healthcare workers as it was precise, easily accessible through a few clicks on their mobile phones and thus could also be revised easily when needed. Although an improvement, particularly in the awareness and some in the participation of the preparedness programs was observed after the intervention, the figures yet remained below 50%. Where this could be ascribed to the growing burden and hectic schedules of the healthcare workers, nonetheless, it signifies the need for methodical and structured management by the administration to take initiatives in educating healthcare workers. The effectiveness of imparting information via WhatsApp and other social media applications is consistent with some of the studies such as the survey done by Oyewole et al. to determine the efficacy of WhatsApp to teach doctors for an exam, and a study conducted in Saudi Arabia in which social media was used by diabetics to communicate about the disease (13, 14).

Three major government hospitals of Rawalpindi were included in the study rather than a single institution in order to make sure that the results held external validity as well. In Pakistan, government hospitals are visited by a larger number of people as compared to private establishments, with the majority of the population being poor and uneducated, and subsequently, more likely to be in neglect of precautionary measures, rendering added importance for the healthcare professionals to be knowledgeable and prepared (15, 16). A part of the pre-intervention study was focused on finding out a statistical significance between the doctors, nurses and the non-clinical hospital staff, the results of which revealed that the non-clinical workforce (that included janitors, security workers, technicians, ward clerks, etc.), which constitutes a fairly significant proportion of the hospital community, were far less informed compared to the doctors and the nurses, in most of the categories. A survey done in Singapore found out that the non-clinical workers were at a higher risk of psychological distress due to COVID-19 than physicians and nurses, the possible reason for which was stated as a lack of medical knowledge on the pandemic, which this audit cycle found out to be true (17). The finding reflects for the educational focus to be shifted on such working staff as well, as they not only carry out a major deal of the interdepartmental work, but also move around in the hospital to a significant degree, and thus their lack of preparedness for the disease can be a major source of spread and possible morbidity. The results also indicated that a greater number of nurses and the supporting non-clinical staff had dealt with an outbreak previously in comparison with the doctors, which is because the doctors working in the frontline are juniors, with a maximum of 5 years of experience under their belt. A larger fraction of the nurses complied with source control measures in contrast to the doctors who were following them the least among the three groups. The majority of healthcare professionals not observing these measures gave the reason to be a shortage of masks. Although an assumption could be made that one group could be changing its masks more or less often than what the guidelines from reliable sources indicate, further exploration into the matter is advised.

After the intervention, the participants considered themselves better prepared to deal with the COVID-19 situation which is particularly fruitful in the scenario because an increase in perceived self-confidence can result in better workplace performance (18). Additionally, after the intervention, the healthcare workers were significantly better informed about the patients needed to be investigated further (PUI) and about the diagnostic investigation, which can avoid a large number of unnecessary testing. There was also a significant improvement in their knowledge about correct methods to make use of Personal Protective Equipment (PPE) which would automatically minimize their chances of getting infected themselves (19). As PPE and testing kits are already in shortage in Pakistan, better knowledge on these measures will not only be able to make appropriate use of available resources but also cut costs and reduce the growing economic burden on a large scale (20–22). Thus, interventions like these may facilitate local implementation of international clinical guidance that ultimately may lead to flattening the curve.

There are a few limitations of the study as well. The lab personnel were excluded from the study since CDC has separate additional infection prevention and control guidelines for them that were not explored by us (23). The questionnaire was translated in Urdu for those individuals who couldn't understand English, which could have resulted in some degree of inevitable disparity. Moreover, the audit was re-conducted 2 weeks later. A shorter duration was kept in accordance with the rapid exponential rise in the number of cases and therefore, the aim was to explore the measures which could be employed to bring improvement as soon as possible. Additionally, the duty stations are rotated after every few weeks among the teams, hence the questionnaire was repeated such to diminish any temporary effect that may have resulted.

The foundation of the intervention was based on an informative self-learning method, which may not have been ideal but in order to comply with the protocol of social distancing to limit transmission, options were limited. Therefore, workshops, classes, and lectures with face-to-face teaching could not be organized. Furthermore, there was a chance of limited involvement of the participants with online meetings, especially since the work overload had increased in the already hectic schedules of the medical community owing to the pandemic, which is why they were not conducted. Further studies are advised to establish whether the preparedness could be further improved through combined approaches of clinical training and didactic teaching ways. Finally, the study alone is not able to confirm if an actual change in the management and practice was continued over time. Hence, the official inclusion of such guidelines is imperative.

## Conclusions

Since the COVID-19 pandemic has spread into various regions of the world, most of the countries, including the developed states of the United States of America, China, Italy, have had an enormous burden placed on their health care systems; physical, mental and financial. The disease is new and much about it is unknown and thus, health organizations all over the world are developing guidelines as they discover more on how healthcare professionals need to be prepared accordingly. The audit conducted indicated that the doctors and the nurses had a better performance in answering most of the knowledge questions but the practice of precautionary methods was still inadequate. The non-clinical staff, however, still was lacking in most aspects, and considering that a healthcare facility cannot function without them, it is the need of the hour to educate and prepare them accordingly. Moreover, after the information from reliable sources was distributed via social media platforms, a statistically significant improvement in performance was observed, which allows the authors to establish that official planning and strategies to ensure the spread of accurate information and the correct methods to make use of safety measures is crucial.

## Data Availability Statement

The raw data supporting the conclusions of this article will be made available by the authors, without undue reservation.

## Ethics Statement

The studies involving human participants were reviewed and approved by Research and Ethical Committee, Rawalpindi Medical University and Allied Hospitals, Rawalpindi. The patients/participants provided their written informed consent to participate in this study.

## Author Contributions

NZ developed the original idea and structured the manuscript. ZJ compiled the data and managed the final formatting. NZ and ZJ performed the statistical analysis and interpretation. MM carried out the proofreading. All authors have agreed to the submission process.

## Conflict of Interest

The authors declare that the research was conducted in the absence of any commercial or financial relationships that could be construed as a potential conflict of interest.
